# Associations of lipid parameters with non-alcoholic fatty liver disease in type 2 diabetic patients according to obesity status and metabolic goal achievement

**DOI:** 10.3389/fendo.2022.1002099

**Published:** 2022-09-16

**Authors:** Zengzhe Zhu, Ningning Yang, Hongmei Fu, Gang Yuan, Yong Chen, Tingting Du, Xinrong Zhou

**Affiliations:** ^1^ Department of Endocrinology, Tongji Hospital, Tongji Medical College, Huazhong University of Science and Technology, Wuhan, China; ^2^ Branch of National Clinical Research Center for Metabolic Diseases, Wuhan, China; ^3^ Department of Endocrinology, Lu’an Hospital of Anhui Medical University, Lu'an, China; ^4^ Department of Geriatrics, Pu’er People’s Hospital, Pu'er, China; ^5^ Laboratory of Endocrinology, Tongji Hospital, Huazhong University of Science and Technology, Wuhan, China

**Keywords:** non-alcoholic fatty liver disease, diabetes mellitus, obesity, blood pressure, lipids

## Abstract

**Aims:**

Non-obese non-alcoholic fatty liver disease (NAFLD) phenotype has sparked interest and frequently occurred in type 2 diabetes mellitus (T2DM). Information on associations between lipid parameters and NAFLD in non-obese patients with diabetes has been lacking. We aimed to investigate the relationships between lipid parameters and NAFLD according to obesity status and metabolic goal achievement in T2DM patients.

**Methods:**

A total of 1,913 T2DM patients who were hospitalized between June 2018 and May 2021 were cross-sectionally assessed. We used logistic regression models to estimate the associations of lipid parameters with NAFLD risk according to obesity and metabolic goal achievement status.

**Results:**

Higher triglycerides, non-HDL-cholesterol, and all lipid ratios including (total cholesterol/HDL-cholesterol, triglyceride/HDL-cholesterol, LDL-cholesterol/HDL-cholesterol, non-HDL-cholesterol/HDL-cholesterol), and lower HDL-cholesterol were associated with NAFLD risk in both non-obese and obese patients. The associations were stronger in non-obese patients than in obese patients. Further, the inverse associations of total cholesterol and LDL-cholesterol with NAFLD risk were only detected in non-obese patients. Triglycerides, HDL-cholesterol, and all lipid ratios studied were significantly associated with NAFLD risk, irrespective of whether the patients achieved their HbA1c, blood pressure, and LDL-cholesterol goal. The presence of poor lipids and lipid ratios were more strongly associated with NAFLD in patients who attained the HbA1c, blood pressure, and/or LDL-cholesterol goal than in those who did not achieve the goal attainment.

**Conclusions:**

The associations of lipids and lipid ratios with NAFLD risk were stronger in T2DM patients who were non-obese and achieved the HbA1c, blood pressure, and/or LDL-cholesterol goal attainment.

## Introduction

Non-alcoholic fatty liver disease (NAFLD) has become one of the major liver diseases worldwide, affecting around 25.2% of the global population (1). It may surpass alcohol as the leading cause for liver transplantation (1). The NAFLD epidemic has paralleled that of the diabetes epidemic. Approximately 60-70% of patients with type 2 diabetes mellitus (T2DM) suffered from NAFLD (2). T2DM is an aggravating factor for NAFLD. For example, it was reported that T2DM patients were at 2 to 4-fold risk for developing advanced liver fibrosis, cirrhosis, liver failure, and hepatocellular carcinoma compared to those without T2DM (3); Vice versa, patients with NAFLD are more commonly progress toward diabetic micro- and macro-vascular complications (4).

Dyslipidemia plays a central role in the pathogenesis of NAFLD (5, 6). Accumulating evidence showed that lipid profile was significantly associated with an increased risk of NAFLD in the general population (7–9). Insulin resistance (IR), well known in T2DM and the main physio-pathological link between NAFLD and T2DM (10–12), triggers an increase in free fatty acids from peripheral adipose tissue and favoring the development of dyslipidemia. However, whether lipids can affect NAFLD independent of IR in T2DM is less well-defined. Additionally, despite NAFLD is predominantly seen with overweight or obesity, this entity can occur in non-obese individuals (13). It was reported that the global prevalence of non-obese NAFLD was above 40% among the NAFLD population and nearly 20% in non-obese population (14). Non-obese NAFLD can develop IR and the full spectrum of metabolic comorbidities and liver damage that occurs in obese NAFLD (13, 15) and may have as severe consequences as obese NAFLD (16). Previous studies conducted in general population have shown that the association between dyslipidemia and NAFLD was more pronounced in non-obese persons than in obese persons (17). It is unclear whether lipid parameters play a role in non-obese T2DM patients and whether the associations between lipid parameters and NAFLD differ between non-obese and obese patients with diabetes. Further, NAFLD is more frequent in patients with poor “ABCs” (parameters usually followed by clinicians for diabetes control, including glycated hemoglobin [HbA1c] [A], blood pressure [BP] [B], and low-density lipoprotein cholesterol [LDL-C] [C]) metabolic treatment goals. It remains unclear whether lipid parameters are associated with different risks of NAFLD in distinct populations defined by glycated hemoglobin (HbA1c), blood pressure (BP), and low-density lipoprotein cholesterol (LDL-C) levels. Therefore, we aimed to investigate the relationships between lipid variables and NAFLD according to obesity and metabolic treatment goal status in T2DM.

## Methods

### Study design and population

This cross-sectional study included 2,946 T2DM patients hospitalized in the Department of Endocrinology, Tongji Hospital, Tongji medical college, Huazhong University of Science and Technology (Wuhan, China) between June 2018 and May 2021. T2DM was diagnosed according to the 2022 American Diabetes Association criteria (18). The exclusion criteria included a history of alcohol abuse (alcohol consumption >140 g/week for male or >70 g/week for female), other causes of hepatic diseases including viral hepatitis, autoimmune liver disease and cirrhosis, current diagnosis of life-threatening cancer, severe psychiatric disturbance, pregnancy or lactation. We excluded 516 with alcohol abuse, 145 with viral hepatitis, and 1 with hepatic cirrhosis; 127 with missing data on blood lipids and liver ultrasound. In addition, to avoid the effects of lipid-lowering on all lipid parameters, 244 participants with lipid-lowering medication use were excluded. The remaining 1,913 subjects were included in our data analyses According to the Private Information Protection Law, information that might identify subjects was safeguarded by the Computer Center. This study was approved by the institutional review board of Tongji Hospital. Because we only retrospectively accessed a de-identified database for purposes of analyses, informed consent requirement was exempted by the institutional review board.

### Clinical measurements

Patients’ data including age, sex, height, weight, current and previous illness histories, and medical treatments were obtained from medical records. Weight was measured with participants wearing light clothing on a calibrated beam scale. Height was measured without shoes. Waist circumference (WC) was measured with an inelastic tape at a midpoint between the bottom of the rib cage and the top of the iliac crest at the end of exhalation. Seated systolic/diastolic BP was measured in triplicate after a 10-min rest, using mercury manometers. The means of the last two readings was used in data analyses. Body mass index (BMI) was calculated as weight (in kilograms) divided by height in square meters.

Blood was collected from the antecubital vein of each individual after an at least 8-hour overnight fast. Measurements were done soon after the blood samples had been collected, and no samples were stored and reused. Glycated hemoglobin (HbA1c) was measured using high performance liquid chromatography (D‐10™; Bio‐Rad Laboratories, Hercules, CA, USA). Fasting plasma glucose (FPG), triglycerides (TG), total cholesterol (TC), high‐density lipoprotein cholesterol (HDL-C), low‐density lipoprotein cholesterol (LDL-C), alanine aminotransferase (ALT), aspartate aminotransferase (AST), uric acid, and creatinine were measured on an autoanalyzer (Cobas C8000, Roche, Mannheim, Germany). Hepatitis viral antigens/antibodies were detected with corresponding Architect reagents (Architect i2000, Abbott Diagnostics, Abbott Park, IL). Non-HDL-C was calculated as TC minus HDL-C. HOMA-IR was calculated as fasting insulin (μU/mL) × FPG (mmol/L)/22.5.

### Definitions

According to the China Obesity Working Group (19), obesity was defined as BMI≥28kg/m^2^.

Ultrasound tests were performed by certified sonographers using a high-resolution, real-time scanner (model SSD-2000; Aloka Co., Ltd., Tokyo Japan). Certified radiologists used standard criteria in evaluating the presence or absence of hepatic fat. Generally, liver steatosis was defined as the presence of stronger echoes in the hepatic parenchyma compared with echoes in the kidney or spleen parenchyma (20). The presence of advanced liver fibrosis was defined as the presence of the high probability for advanced fibrosis calculated by NAFLD fibrosis score (NFS) or BARD score. NFS was calculated as -1.675 + 0.037 × age (years) + 0.094 × BMI (kg/m^2^) + 1.13 × IFG/diabetes (yes 1, no 0) + 0.99 × AST/ALT ratio - 0.013 × platelet (10^9^/L) - 0.66 × albumin (g/dl) (21). The presence of advanced liver fibrosis was confirmed when the score was greater than 0.676. BARD score: BMI > 28 = 1 point, AAR (Aspartate transaminase/alanine animo-transferase [AST/ALT] ratio) of > 0.8 = 2 points, DM = 1 point. A score of ≥ 2 was associated with advanced fibrosis (22).

### Statistical analyses

All statistical analyses were conducted using SPSS software (version 24.0 for mac; SPSS, Chicago, IL, USA). Continuous variables were presented as means (minimum to maximum) or medians (IQRs) depending on their distribution. Categorical variables were presented as percentages. Differences in continuous variables between groups were tested with one-way ANOVA or Kruskal-Wallis test. Differences in categorical variables were tested with χ^2^ test. Logistic regression models were used to estimate the associations (odds ratios [ORs], with 95% confidence Intervals [CIs]) between each lipid parameter and risk of NAFLD. Four models were fitted. Model 1 was adjusted for age, smoking status, family history of diabetes. Model 2 was additionally adjusted for body mass index, systolic blood pressure, glycated hemoglobin, use of anti-hypertensive drug. Model 3 was additionally adjusted for HOMA-IR. Model 4 was additionally adjusted for anti-diabetic drug use. A receiver operating characteristic (ROC) curve analysis was performed for each lipid parameter to compare the abilities of these measures to discriminate NAFLD correctly. The overall diagnostic accuracy was quantified using the area under the ROC curve (AUC). Significance was accepted at a two-tailed P <0.05.

## Results

### Baseline characteristics of study subjects

Of the 1,913 T2DM patients included in the present analyses, the mean age was 52.1 (13.3) years, 55.2% were men, the mean BMI value was 24.9 (3.8) kg/m^2^. The overall prevalence of NAFLD was 48.5%. 73.49% diabetic patients with NAFLD were non-obese. T2DM patients with obese NAFLD phenotype have a mean BMI value of 31.14 (3.33) kg/m^2^ and a mean HbA1c value of 9.86% (2.36%). The corresponding figures were 22.92 (2.68) kg/m^2^ and 9.14% (2.57%), respectively, for T2DM patients with non-obese NAFLD phenotype. As seen in **Table 1**, NAFLD patients were younger, had higher BMI, WC, HbA1c, AST, ALT and adverse lipids and lipid ratios than patients without NAFLD (all P value <0.001). Moreover, NAFLD patients were less likely to have the care goal achievement (all P value <0.001).

**Table 1:** Characteristics of participants according to non-alcoholic fatty liver disease status.

	Without NAFLD	With NAFLD	P value
n	985	928	
Male, %	55.20	60.23	0.011
Smoking, %	17.25	19.94	0.084
Age, years	55.55 (14-85)	50.23 (14-89)	<0.001
Weight, kg	63.98 (30-105)	73.62 (45-159.3)	<0.001
BMI, kg/m^2^	23.60 (15.31-37.46)	26.38 (18.75-49.63)	<0.001
Obesity, %	16.94	26.51	<0.001
WC, cm	89.20 (62-129)	95.86 (74-188)	<0.001
SBP, mmHg	130.70 (70-216)	132.08 (76-215)	0.106
DBP, mmHg	80.09 (49-137)	84.22 (46-133)	<0.001
HbA1c, %	9.05 (4.30-18.10)	9.74 (5.20-18.70)	<0.001
ALT, U/L	21.27 (5-450)	32.47 (5-393)	<0.001
AST, U/L	20.01 (5-212)	25.15 (5-317)	<0.001
TC, mmol/L	4.38 (1.78-13.70)	4.76 (1.56-14.10)	<0.001
TG, mmol/L	2.15 (0.36-22.06)	3.60 (0.21-45.21)	<0.001
HDL-C, mmol/L	1.12 (0.23-2.82)	0.97 (0.38-2.03)	<0.001
LDL-C, mmol/L	2.68 (0.61-7.14)	2.82 (0.20-6.23)	<0.001
TC/HDL-C	4.15 (1.59-18.92)	5.16 (1.89-32.05)	<0.001
TG/HDL-C	2.27 (0.21-36.77)	4.30 (0.24-88.65)	<0.001
LDL/HDL-C	2.53 (0.48-7.61)	2.98 (0.38-10.02)	<0.001
non-HDL-C, mmol/L	3.26 (0.94-12.56)	3.79 (1.00-13.66)	<0.001
nonH-DL-C/HDL-C	3.15 (0.59-17.92)	4.16 (0.89-31.05)	<0.001
Anti-hypertensive drug use, %	32.28	31.79	0.816
Anti-diabetic drug use			
Sulfonylureas use, %	14.98	17.97	0.078
Non-sulfonylureas use, %	1.94	3.15	0.095
Metformin use, %	30.82	32.59	0.589
Glucosidase inhibitor use, %	18.43	28.73	<0.001
Thiazolidinediones use, %	5.17	7.92	0.016
DPP4i use, %	6.25	7.72	0.209
SGLT2i use, %	3.02	3.45	0.592
Insulin use, %	22.31	39.70	<0.001
GLP-1 RA use, %	1.72	0.81	0.073
‘A’ attained, %	14.72	5.93	<0.001
‘B’ attained, %	35.63	25.54	<0.001
‘C’ attained, %	46.80	37.82	<0.001

Values are proportions, and means (minimum to maximum)

BMI, body mass index; WC, waist circumference; SBP, systolic blood pressure; DBP, diastolic blood pressure; HbA1c, glycated hemoglobin; ALT, alanine animo-transferase; AST, aspartate transaminase; TC, total cholesterol; TG, triglycerides; HDL-C, high density lipoprotein cholesterol; LDL-C, low density lipoprotein cholesterol; DPP4i, Dipeptidyl peptidase-4 inhibitor; SGLT2i, Sodium-glucose cotransporter 2 inhibitor; GLP-1 RA, glucagon-like peptide 1 receptor agonists; ‘A’ attained, HbA1c <6.5%; ‘B’ attained, blood pressure < 130/80mmHg; ‘C’ attained, LDL-C <2.6mmol/L.

### ROC analysis of lipids and lipid ratios for identifying NAFLD in patients with diabetes

**Figure 1:**
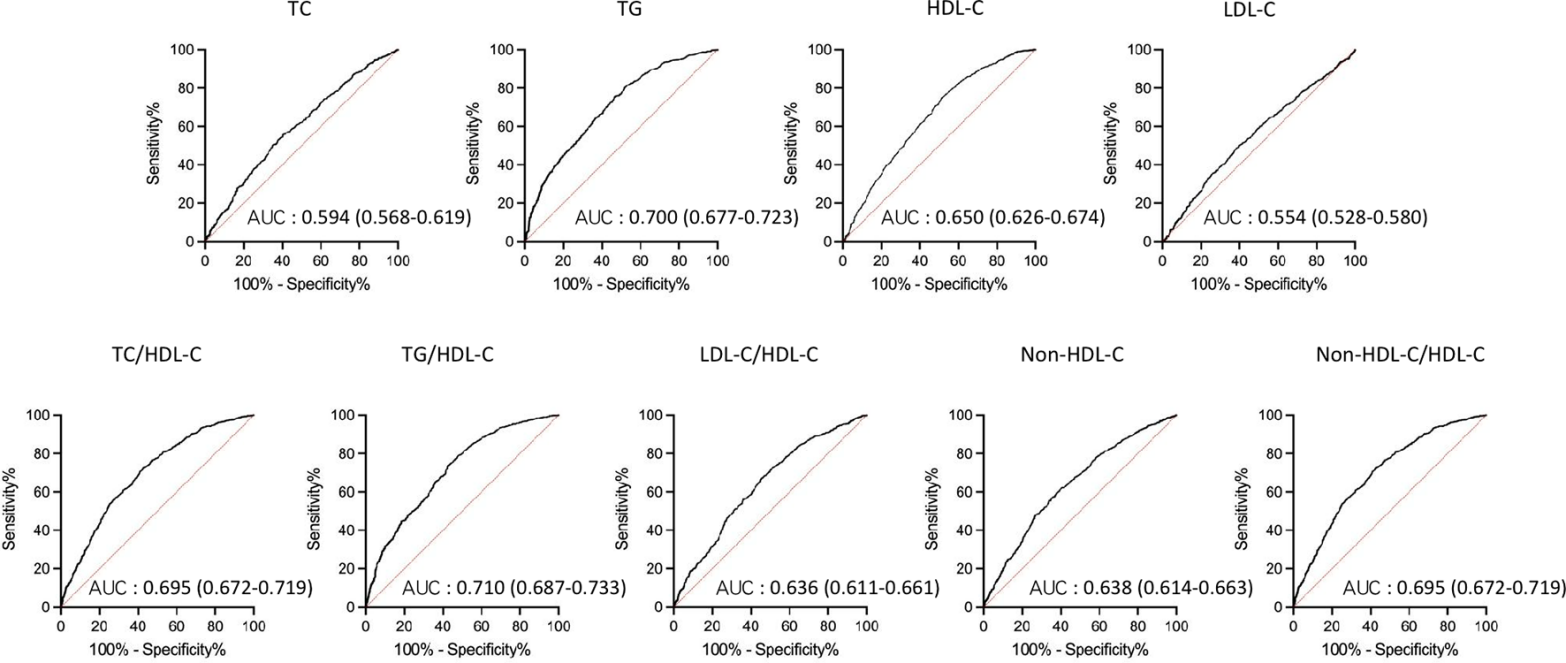
Receiver operating characteristic (ROC) curves of lipid parameters for detecting non-alcoholic fatty liver disease in T2DM patients. AUC, area under the curve; TC, total cholesterol; TG, triglycerides; HDL-C, high density lipoprotein cholesterol; LDL-C, low density lipoprotein cholesterol; T2DM, type 2 diabetes mellitus.

AUCs for all lipid parameters studied indicated that all lipid parameters could effectively discriminate NAFLD (all AUC > 0.5). In addition, AUCs derived from lipid ratios were in general significantly greater than from single lipids (**Figure 1**).

### Associations of lipid parameters with NAFLD according to obesity status

**Figure 2:**
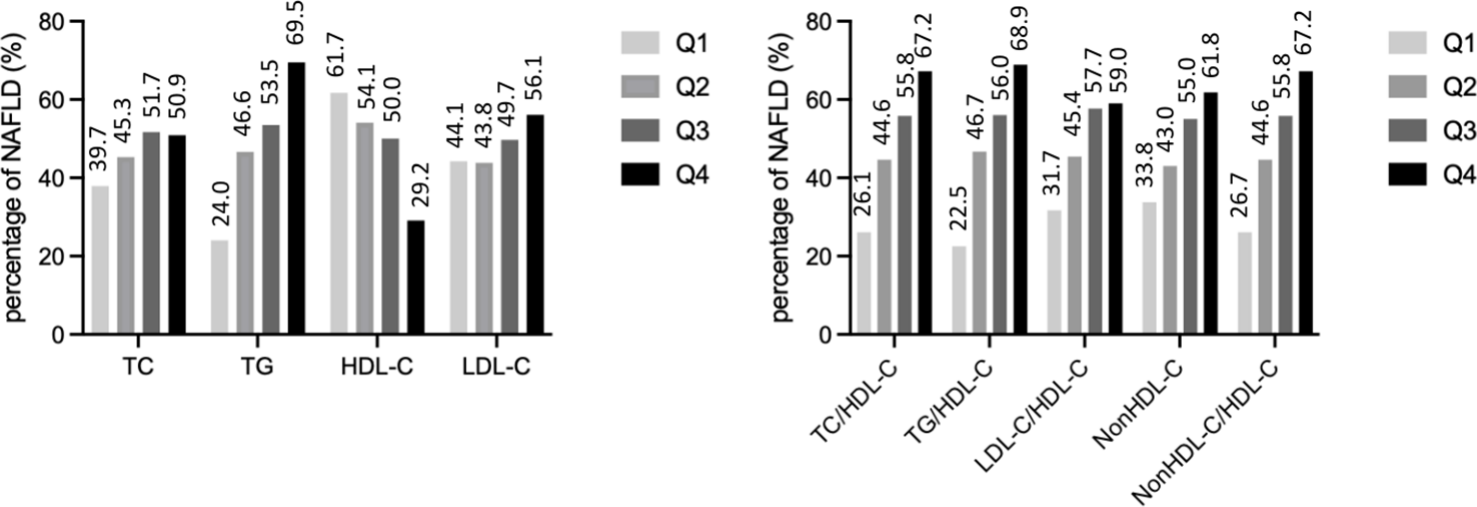
The prevalence of non-alcoholic fatty liver disease by quartiles of lipid parameters. TC, total cholesterol; TG, triglycerides; HDL-C, high density lipoprotein cholesterol; LDL-C, low density lipoprotein cholesterol.

The prevalence of NAFLD increased from the first to the fourth quartiles of the serum TG levels and each lipid ratio and decreased from the first to fourth quartiles of the serum HDL-C levels (all P <0.001) (**Figure 2**).

The associations of lipid parameters with NAFLD according to obesity status were shown in **Table 2**. After controlling for potential intermediate variables including HOMA-IR and anti-diabetic medication use, all lipid parameters studied, except LDL-C, were significantly associated with NAFLD in non-obese T2DM patients. Among obese T2DM subjects, TG and each lipid ratio were positively associated with NAFLD, while HDL-C was negatively associated with NAFLD. In both obese and non-obese T2DM patients, lipid ratios were more closely associated with NAFLD than any of the individual variables used alone.

**Table 2:** Odds ratios and 95% confidence intervals of lipid parameters for non-alcoholic fatty liver disease according to obesity status.

	Model	Total	Obese	Non-obese
TC	1	1.28 (1.18-1.38)^*^	1.23 (0.96-1.57)	1.27 (1.17-1.39)^*^
	2	1.22 (1.12-1.33)^*^	1.11 (0.86-1.42)	1.28 (1.16-1.41)^*^
	3	1.23 (1.12-1.35)^*^	1.13 (0.88-1.47)	1.28 (1.15-1.43)^*^
	4	1.22 (1.11-1.34)^*^	1.13 (0.87-1.46)	1.25 (1.12-1.39)^*^
TG	1	1.29 (1.22-1.36)^*^	1.26 (1.06-1.48)^#^	1.27 (1.20-1.35)^*^
	2	1.22 (1.15-1.28)^*^	1.22 (1.02-1.45)^#^	1.21 (1.14-1.29)^*^
	3	1.21 (1.15-1.28)^*^	1.26 (1.06-1.51)^#^	1.19 (1.13-1.27)^*^
	4	1.21 (1.15-1.28)^*^	1.23 (1.04-1.46)^#^	1.19 (1.12-1.26)^*^
HDL-C	1	0.15 (0.11-0.22)^*^	0.07 (0.02-0.25)^*^	0.19 (0.13-0.28)^*^
	2	0.24 (0.16-0.36)^*^	0.10 (0.03-0.33)^*^	0.34 (0.22-0.52)^*^
	3	0.22 (0.14-0.34)^*^	0.08 (0.02-0.31)^*^	0.30 (0.19-0.49)^*^
	4	0.23 (0.15-0.36)^*^	0.10 (0.02-0.38)^*^	0.28 (0.17-0.46)^*^
LDL-C	1	1.18 (1.07-1.30)^#^	1.19 (0.89-1.58)	1.20 (1.07-1.33)^*^
	2	1.13 (1.01-1.26)^#^	1.05 (0.76-1.44)	1.16 (1.03-1.31)^#^
	3	1.12 (1.00-1.27)	1.05 (0.76-1.47)	1.15 (1.01-1.31)^#^
	4	1.08 (0.96-1.22)	1.04 (0.75-1.45)	1.09 (0.95-1.25)
TC/HDL-C	1	1.52 (1.41-1.64)^*^	1.58 (1.26-1.99)^*^	1.48 (1.36-1.60)^*^
	2	1.37 (1.27-1.49)^*^	1.37 (1.09-1.73)^#^	1.34 (1.23-1.47)^*^
	3	1.39 (1.27-1.51)^*^	1.47 (1.16-1.86)^*^	1.38 (1.26-1.51)^*^
	4	1.39 (1.28-1.51)^*^	1.41 (1.11-1.79)^*^	1.32 (1.20-1.45)^*^
TG/HDL-C	1	1.20 (1.15-1.25)^*^	1.22 (1.02-1.42)^#^	1.18 (1.13-1.23)^*^
	2	1.14 (1.10-1.19)^*^	1.18 (1.02-1.35)^#^	1.13 (1.08-1.18)^*^
	3	1.14 (1.09-1.19)^*^	1.22 (1.05-1.41)^#^	1.12 (1.07-1.17)^*^
	4	1.14 (1.10-1.19)^*^	1.20 (1.04-1.40)^#^	1.12 (1.07-1.17)^*^
LDL-C/HDL-C	1	1.52 (1.37-1.67)^*^	1.64 (1.23-2.22)^*^	1.49 (1.34-1.65)^*^
	2	1.36 (1.22-1.51)^*^	1.40 (1.03-1.91)^#^	1.31 (1.17-1.47)^*^
	3	1.37 (1.22-1.53)^*^	1.40 (1.03-1.92)^#^	1.31 (1.16-1.48)^*^
	4	1.34 (1.20-1.51)^*^	1.38 (1.01-1.81)^#^	1.27 (1.12-1.44)^*^
Non-HDL-C	1	1.45 (1.32-1.58)^*^	1.40 (1.08-1.82)^#^	1.43 (1.30-1.58)^*^
	2	1.34 (1.22-1.47)^*^	1.21 (0.92-1.57)	1.40 (1.25-1.55)^*^
	3	1.35 (1.22-1.50)^*^	1.24 (0.94-1.63)	1.40 (1.25-1.57)^*^
	4	1.34 (1.21-1.48)^*^	1.23 (0.93-1.61)	1.36 (1.21-1.52)^*^
Non-HDL-C/HDL-C	1	1.52 (1.41-1.64)^*^	1.58 (1.26-1.99)^*^	1.48 (1.36-1.60)^*^
	2	1.37 (1.27-1.49)^*^	1.36 (1.07-1.74)^#^	1.34 (1.23-1.47)^*^
	3	1.39 (1.27-1.51)^*^	1.47 (1.16-1.86)^*^	1.38 (1.26-1.51)^*^
	4	1.39 (1.28-1.51)^*^	1.41 (1.11-1.79)^*^	1.32 (1.20-1.45)^*^

Model 1 was adjusted for age, sex, smoking status, family history of diabetes mellitus.

Model 2 was adjusted for all the variables in model 1 plus SBP, BMI, HbA1c and use of anti-hypertensive drugs for total; In obesity and non-obesity subgroup, BMI was replaced by waist circumference.

Model 3 was adjusted for all the variables in model 2 plus HOMA-IR.

Model 4 was adjusted for all the variables in model 3 plus use of anti-diabetic drugs.

TC, total cholesterol; TG, triglycerides; HDL-C, high density lipoprotein cholesterol; LDL-C, low density lipoprotein cholesterol; SBP, systolic blood pressure; BMI, body mass index; HbA1c, glycated hemoglobin.

^*^P < 0.001, ^#^P < 0.05.

The odds ratios (ORs) and 95% confidence intervals (CIs) of quartiles of each lipid parameter for NAFLD were presented in **Table 3**. Among both non-obese and obese patients, after controlling for potential intermediate variables including HOMA-IR and anti-diabetic medication use, higher TG, TC/HDL-C, TG/HDL-C, and non-HDL-C/HDL-C, and lower HDL-C were significantly associated with NAFLD risk. In non-obese subjects, higher TC, LDL-C, non-HDL-C, and LDL-C/HDL-C levels were also significantly associated with NAFLD risk.

**Table 3:** Odds raitos and 95% confidence intervals of lipid parameters in terms of the quartiles for non-alcoholic fatty liver disease according to obesity status.

		Total		Obese		Non-obese	
		OR (95% CI)	P	OR (95% CI)	P	OR (95% CI)	P
TC	Q1	ref.		ref.		ref.	
	Q2	1.10 (0.80-1.51)	0.572	0.84 (0.37-0.93)	0.683	1.09 (0.76-1.57)	0.639
	Q3	1.51 (1.09-2.10)	0.013	1.88 (0.74.4.61)	0.170	1.50 (1.05-2.16)	0.028
	Q4	1.78 (1.24-2.48)	0.001	1.62 (0.64-4.06)	0.473	1.73 (1.20-2.50)	0.004
TG	Q1	ref.		ref.		ref.	
	Q2	2.08 (1.50-2.89)	<0.001	1.64 (0.73-3.66)	0.228	2.61 (1.78-3.84)	<0.001
	Q3	2.64 (1.90-3.68)	<0.001	4.67 (1.89-10.50)	0.001	2.89 (1.97-4.26)	<0.001
	Q4	4.73 (3.35-6.68)	<0.001	4.68 (1.66-13.22)	0.004	4.99 (3.37-7.39)	<0.001
HDL-C	Q1	ref.		ref.		ref.	
	Q2	0.69 (0.50-0.96)	0.028	1.29 (0.42-3.91)	0.656	0.73 (0.52-1.04)	0.081
	Q3	0.60 (0.43-0.82)	0.002	0.72 (0.27-1.90)	0.503	0.69 (0.49-0.98)	0.038
	Q4	0.32 (0.23-0.46)	<0.001	0.41 (0.15-1.08)	0.071	0.39 (0.27-0.58)	<0.001
LDL-C	Q1	ref.		ref.		ref.	
	Q2	1.16 (0.84-1.59)	0.374	1.16 (0.50-2.71)	0.735	1.07 (0.75-1.53)	0.697
	Q3	1.14 (.083-1.56)	0.427	1.40 (0.57-3.42)	0.464	1.11(0.78-1.57)	0.572
	Q4	1.38 (1.00-1.90)	0.050	1.30 (0.54-3.15)	0.557	0.41 (0.99-2.02)	0.058
TC/HDL-C	Q1	ref.		ref.		ref.	
	Q2	1.84 (1.33-2.55)	0.002	3.93 (1.72-8.99)	0.001	1.60 (1.09-2.34)	0.016
	Q3	2.55 (1.83-3.57)	<0.001	3.64 (1.44-9.22)	0.006	2.47 (1.70-3.59)	<0.001
	Q4	3.23 (2.28-4.57)	<0.001	3.33 (1.26-8.78)	0.015	3.36 (2.28-4.94)	<0.001
TG/HDL-C	Q1	ref.		ref.		ref.	
	Q2	2.67 (1.92-3.72)	<0.001	1.52 (0.69-3.36)	0.297	3.02 (2.04-4.46)	<0.001
	Q3	2.76 (1.98-3.85)	<0.001	4.93 (1.95-12.44)	0.001	2.94 (1.99-4.35)	<0.001
	Q4	5.25 (3.69-7.50)	<0.001	5.12 (1.65-15.88)	0.005	5.14 (3.45-7.68)	<0.001
LDL-C/HDL-C	Q1	ref.		ref.		ref.	
	Q2	1.74 (1.26-2.40)	0.001	2.20 (0.94-5.15)	0.069	1.74 (1.20-2.51)	0.003
	Q3	2.15 (1.55-2.96)	<0.001	2.37 (1.01-5.53)	0.047	2.14 (1.49-3.06)	<0.001
	Q4	1.97 (1.41-2.75)	<0.001	1.35 (0.54-3.38)	0.518	1.83 (1.26-2.66)	0.001
Non-HDL-C	Q1	ref.		ref.		ref.	
	Q2	1.42 (1.02-1.97)	0.037	0.94 (0.42-2.13)	0.886	1.29 (0.89-1.87)	0.176
	Q3	2.26 (1.62-3.12)	<0.001	2.07 (0.84-5.15)	0.116	1.90 (1.32-2.74)	0.001
	Q4	2.44 (1.75-3.41)	<0.001	1.96 (0.69-4.48)	0.236	2.27 (1.57-3.29)	<0.001
Non-HDL/HDL-C	Q1	ref.		ref.		ref.	
	Q2	1.69 (1.21-2.35)	0.002	3.93 (1.72-8.99)	0.001	1.60 (1.09-2.34)	0.016
	Q3	2.55 (1.83-3.57)	<0.001	3.64 (1.44-9.22)	0.006	2.47 (1.70-3.59)	<0.001
	Q4	3.23 (2.28-4.57)	<0.001	3.33 (1.26-8.78)	0.015	3.36 (2.28-4.94)	<0.001

Odds ratios were adjusted for age, sex, smoking status, family history of diabetes mellitus, SBP, BMI, HbA1c, HOMA-IR, use of anti-hypertensive drugs, and anti-diabetic drugs.

TC, total cholesterol; TG, triglycerides; HDL-C, high density lipoprotein cholesterol; LDL-C, low density lipoprotein cholesterol; SBP, systolic blood pressure; BMI, body mass index; HbA1c, glycated hemoglobin.

### Odds ratios of lipid parameters for NAFLD according to diabetes control parameters

The associations of lipid parameters with NAFLD in different T2DM control parameters, namely HbA1c (A), BP (B), and LDL-C (C) were shown in **Table 4**. After adjusting for potential confounding variables, TG, HDL-C, and all lipid ratios studied were significantly associated with NAFLD risk, irrespective of A, B, and C status. The associations of lipid parameters with NAFLD were stronger in patients who achieved the A, B, and/or C goal. Moreover, lipid ratios were more closely associated with NAFLD risk than any of the individual variables used alone, regardless of whether the patients reached their care goal attainment.

**Table 4:** Odds ratios 95% confidence intervals of lipid parameters for non-alcoholic fatty liver disease according to metabolic goal attainment status.

	HbA1c ≥ 6.5%	HbA1c < 6.5%	BP ≥ 130/80mmHg	BP <130/80mmHg	LDL-C ≥ 2.6mmol/L	LDL-C < 2.6mmol/L
TC	1.19 (1.07-1.33)^*^	1.46 (0.85-2.49)	1.22 (1.08-1.37)^*^	1.20 (1.03-1.41)^#^	1.19 (1.02-1.38)^#^	1.29 (1.10-1.52)^*^
TG	1.23 (1.15-1.31)^*^	1.53 (1.09-2.15)^#^	1.31 (1.21-1.42)^*^	1.11 (1.04-1.19)^#^	1.34 (1.22-1.51)^*^	1.18 (1.11-1.25)^*^
HDL-C	0.25 (0.15-0.41)^*^	0.02 (0.002-0.15)^*^	0.18 (0.10-0.31)^*^	0.36 (0.16-0.80)^#^	0.29 (0.15-0.53)^*^	0.10 (0.05-0.21)^*^
LDL-C	1.07 (0.93-1.23)	2.14 (1.02-4.48)^#^	1.02 (0.88-1.18)	1.22 (0.97-1.53)	1.10 (0.88-1.37)	0.98 (0.67-1.45)
TC/HDL-C	1.37 (1.24-1.51)^*^	2.62 (1.58-4.36)^*^	1.49 (1.34-1.65)^*^	1.24 (1.10-1.41)^*^	1.37 (1.21-1.55)^*^	1.39 (1.24-1.57)^*^
TG/HDL-C	1.16 (1.10-1.22)^*^	1.69 (1.23-1.33)^*^	1.22 (1.15-1.30)^*^	1.08 (1.02-1.13)^*^	1.31 (1.20-1.44)^*^	1.12 (1.08-1.17)^*^
LDL-C/HDL-C	1.29 (1.11-1.47)^*^	3.90 (1.88-8.10)^*^	1.32 (1.15-1.52)^*^	1.32 (1.08-1.63)^#^	1.33 (1.13-1.57)^*^	1.62 (1.27-2.07)^*^
Non-HDL-C	1.29 (1.15-1.45)^*^	1.65 (1.00-2.71)^#^	1.36 (1.20-1.55)^*^	1.26 (1.07-1.48)^*^	1.30 (1.11-1.53)^*^	1.48 (1.23-1.78)^*^
Non-HDL-C/HDL-C	1.37 (1.24-1.51)^*^	2.62 (1.58-4.36)^*^	1.49 (1.34-1.65)^*^	1.24 (1.10-1.41)^*^	1.37 (1.21-1.55)^*^	1.39 (1.24-1.57)^*^

Model for HbA1c subgroup was adjusted for age, sex, smoking status, family history of diabetes mellitus, SBP, BMI, RBG(random blood glucose), HOMA-IR, use of anti-hypertensive drugs, and anti-diabetic drugs;

Model for blood pressure subgroup was adjusted for age, sex, smoking status, family history of diabetes mellitus, BMI, HbA1c, HOMA-IR, use of anti-hypertensive drugs, and anti-diabetic drugs;

Model for LDL-cholesterol subgroup was adjusted for age, sex, smoking status, family history of diabetes mellitus, SBP, BMI, HbA1c, HOMA-IR, use of anti-hypertensive drugs, and anti-diabetic drugs.

TC, total cholesterol; TG, triglycerides; HDL-C, high density lipoprotein cholesterol; LDL-C, low density lipoprotein cholesterol; SBP, systolic blood pressure; BMI, body mass index; HbA1c, glycated hemoglobin; BP, blood pressure.

^*^P < 0.001, ^#^P < 0.05.

### Association of lipid parameters and advanced liver fibrosis

The ORs of quartiles of each lipid parameters for advanced fibrosis, defined by two non-invasive advanced fibrosis predict scores: NFS and BARD, were shown in **Table 5**. Lower HDL-C was significantly associated with advanced fibrosis risk defined by NFS.

**Table 5:** Odds raitos and 95% confidence intervals of lipid parameters in terms of the quartiles for advanced liver fibrosis.

	NFS		BARD		
	≤ 0.676	> 0.676 P value	< 2	≥ 2	P value
TC	ref.	0.95 (0.39-2.28) 0.904	ref.	0.92 (0.62-1.36)	0.668
TG	ref.	0.85 (0.35-2.07) 0.715	ref.	0.89 (0.60-1.32)	0.550
HDL	ref.	4.98 (2.17-11.40) <0.001	ref.	1.25 (0.82-1.90)	0.296
LDL	ref.	0.96 (0.43-2.15) 0.919	ref.	0.91 (0.62-1.34)	0.629
TC/HDL-C	ref.	0.74 (0.28-1.93) 0.534	ref.	1.25 (0.83-1.88)	0.283
TG/HDL-C	ref.	0.58 (0.25-1.36) 0.208	ref.	1.10 (0.74-1.64)	0.637
LDL-C/HDL-C	ref.	0.32 (0.51-3.47) 0.568	ref.	1.04 (0.69-1.55)	0.862
non-HDL	ref.	0.82 (0.34-1.94) 0.648	ref.	1.07 (0.72-1.60)	0.733
non-HDL/HDL-C	ref.	0.43 (0.28-1.93) 0.534	ref.	1.25 (0.83-1.88)	0.283

Odds ratios were adjusted for age, sex, smoking status, family history of diabetes mellitus, SBP, BMI, HbA1c, HOMA-IR, use of anti-hypertensive drugs, and anti-diabetic drugs.

TC, total cholesterol; TG, triglycerides; HDL-C, high density lipoprotein cholesterol; LDL-C, low density lipoprotein cholesterol; SBP, systolic blood pressure; BMI, body mass index; HbA1c, glycated hemoglobin.

### Sensitivity analysis

Since metformin and glucagon-like peptide 1 receptor agonists (GLP-1RAs) are two of the few anti-diabetic medications preventing weight gain or even favoring weight loss in T2DM patients (23, 24), to avoid the effects of these medications use on results, we did the above analysis after excluding patients taking these two drugs. The results were essentially the same (**Supplementary Tables 1, 2**) except in the obese subgroup, in whom the associations were no longer significant. However, estimates in this subgroup should be interpreted with caution due to limited sample size and inadequate statistical power.

## Discussion

This is, as far as we are aware, the first report to describe the associations of lipids and lipid ratios with NAFLD in T2DM patients according to obesity status and metabolic goal achievement status. We found that in patients with T2DM, adverse lipids and lipid ratios were significantly associated with NAFLD risk, regardless of obesity status and metabolic goal attainment status. The associations were stronger in patients who were non-obese and had the A, B, and/or C goal attainment. Moreover, lipid ratios have a stronger association with NAFLD risk than any of the individual variables used alone.

The associations of lipids and lipid ratios with NAFLD have been established in the general population (7–9, 25). However, in patients with T2DM, the associations between lipid parameters and NAFLD risk remain less clear. Here, we verified the significant associations of lipids and lipid ratios with NAFLD risk in T2DM patients. In consistent with previous studies conducted in the general population (7, 8, 25), we also noted that lipid ratios were more effective than single measures of lipids in detecting NAFLD. This may be explained by that lipid ratios taken account of the proportion between the pro-atherogenic and anti-antherogenic fractions (26, 27).

The relatively low BMI in diabetic patients with NAFLD may be due to the following reasons: 1) Chinese individuals are characterized by a greater amount of visceral or ectopic adipose tissue than Europeans at a given BMI (28); 2) Non-obese NAFLD phenotype was more frequent in patients with T2DM. The non-obese NAFLD phenotype has sparked interest because of its high prevalence (6, 7, 29), and unanswered questions regarding whether stratifying NAFLD patients based on their obesity status could prioritize allocation of clinical resources for those most at risk of poor outcomes (30). Reports convinced that non-obese NAFLD subjects had severe impaired glucose tolerance and dyslipidemia that were identical or even worse than obese NAFLD subjects (15, 16). This evidence from general population-based analyses supports that non-obese NAFLD may represent a distinct entity in the disease spectrum of NAFLD. To date, analysis of the association of lipids and lipid ratios with non-obese NAFLD has not been reported in patients with diabetes, in whom NAFLD and dyslipidemia commonly occur (2, 3, 31).

We addressed this fundamental knowledge gap in the present study. We found that more severe dyslipidemias in T2DM, including higher TG, all lipid ratios studied, and lower HDL-C were associated with NAFLD risk in both non-obese and obese patients. The associations were stronger in non-obese patients than in obese patients. Further, the inverse associations of TC and LDL-C levels with NAFLD risk were only detected in non-obese patients. One possible explanation for these results may be due to a decreased capacity for storing fat in adipose tissue in non-obese NAFLD patients (13, 32, 33). According to the overflow hypothesis, adipose tissue acts as a reservoir of free fatty acids and prevents their overflow into insulin-sensitive tissues including liver. Alterations in fatty acid trafficking lead to abnormalities in lipid storage and consequent dyslipidemia and ectopic fat deposition (33). Further, obesity is a well-defined risk factor for NAFLD (34–36). Thus, obesity attenuates the relationship between lipids and lipid ratios and NAFLD. Although the percentage of metformin and/or GLP-1RAs use, which were used for a dual approach of treating both diabetes and NAFLD (23, 24), were similar in T2DM patients with and without NAFLD, to avoid the effects of these medications use on NAFLD, we have adjusted the anti-diabetic medication use. Moreover, we did sensitivity analysis after excluding patients taking these two drugs. The results were essentially the same. This suggested that dyslipidemia in subjects with diabetes, even if they were not obese, might be identified as an indicator of the presence of NAFLD.

Since diabetic control parameters have strong effects on NAFLD and lipid profile (3, 34, 35), we also investigated whether the relationships between lipids and lipid ratios and NAFLD differed by HbA1c, BP, and LDL-C status. We found that in patients with T2DM, TG, HDL-C, and all lipid ratios studied were significantly associated with NAFLD risk, irrespective of A, B, and C status. When further adjusting for the use of anti-diabetic drugs, the results were essentially the same. The presence of poor lipids and lipid ratios were more strongly associated with NAFLD in patients who attained the A, B, and/or C goal than in those who did not achieve the goal attainment. Further, the inverse association of LDL-C levels with NAFLD risk was only detected in patients who achieved the A, B, and/or C goal. One possible explanation for these results may be due to the independent associations of increased HbA1c, BP, and/or LDL-C levels with NAFLD (37–40). The significant and independent associations of lipids and lipid ratios with NAFLD in those who achieved the A, B, and/or C goal attainment highlight that lipids and lipid ratios predispose to increased NAFLD risk, regardless of care goal attainment status. However, estimates across subgroups should be interpreted with caution because of limited sample size and inadequate statistical power.

In the present study, when using NFS and BARD to indicate advanced fibrosis, we found lower HDL-C was significantly associated with advanced fibrosis risk, defined by NFS. The relations between lipid parameters and advanced fibrosis is still controversial (41, 42). Hegazy M,et,al. found that lipid ratios, particularly TG/HDL-C, are associated with advanced fibrosis (43). While other studies showed that the advanced fibrosis risk did not differ by lipid status (44). Further studies are warranted to explore the associations between lipid parameters and advanced fibrosis in T2DM patients.

Our findings have important clinical implications. With the diabetes epidemics in China, the incidence of NAFLD is expected to be even more prevalent in patients with diabetes in the near future. The increased prevalence of NAFLD suggests that more patients with diabetes are predisposed to an increased cardiovascular disease risk. The established insulin resistance (IR) in T2DM plays a key role in the development of NAFLD by increasing the accumulation of free fatty acids in the liver and inhibiting adipose tissue lipolysis (10–12). The current study demonstrates the important impacts of adverse lipids and lipid ratios on NAFLD independent of HOMA-IR in both obese and non-obese T2DM patients. Therefore, NAFLD cannot be explained by IR alone, as other factors such as genetic and epigenetic factors, lipotoxicity, mitochondrial dysfunction, endoplasmic reticulum stress, microbiota, chronic low-grade inflammation and oxidative stress, dysfunction of adipose tissue, and nutritional factors and lifestyle are also involved in the development of the disease (45). Taken together, management of dyslipidemia in patients with T2DM, regardless of obesity status and care goal achievement status, may be therefore of importance for the prevention and reduction of NAFLD and cardiovascular disease risk.

The main strength of this study is the large number of T2DM patients included from an academic hospital. Further, we can get access to clinical, laboratory, and imaging data in medical records, which provided more in-depth clinical information that are not usually available in large epidemiological surveys.

There are several limitations. First, NAFLD was diagnosed by ultrasonography rather than liver histopathology, which may lead to an inaccurate diagnosis. Nevertheless, liver ultrasonography has been confirmed as an accurate and reliable tool for detecting fatty liver. Due to the relatively low cost and lack of radiation exposure, ultrasonography is widely used for identifying fatty liver in clinical settings and population studies. Second, although we adjusted for multiple potential confounding variables, residual and unmeasured confounding might not be fully addressed. Third, our study population were mainly based on inpatients suffering from T2DM, whose health conditions might be severer than those of outpatients. Thus, our findings could not be generalized to outpatients with T2DM. Fourth, the cross-sectional study design makes it difficult to infer causality between the lipid parameters and NAFLD risk. At last, some anti-diabetic drug use in T2DM patients including metformin and/or GLP-RA, can affect weight and liver fat content (23, 24).

In conclusion, in patients with T2DM, lipids and lipid ratios were significantly associated with NAFLD risk, independent of HOMA-IR, irrespective of obesity status and metabolic goal attainment status. The associations of lipids and lipid ratios with NAFLD risk were stronger in T2DM patients who were non-obese and achieved the HbA1c, blood pressure, and/or LDL-cholesterol goal attainment.

## Data availability statement

The raw data supporting the conclusions of this article will be made available by the authors, without undue reservation.

## Ethics statement

This study was approved by the institutional review board of Tongji Hospital. Because we only retrospectively accessed a de-identified database for purposes of analysis, informed consent was not required.

## Author contributions

ZZ and NY, study design, statistical analyses, acquisition and interpreting of data, and drafting of manuscript. HF, acquisition of data. GY and YC, critical revision of the manuscript. TD and XZ, study design, interpreting data, and critical revision of the manuscript. All authors contributed to the article and approved the submitted version.

## Funding

This study was supported by the National Natural Science Foundation of China (82070859 to YC) and Bethune Charitable Foundation 2020 (to TD).

## Acknowledgments

The authors thank all study participants for their participation.

## Conflict of interest

The authors declare that the research was conducted in the absence of any commercial or financial relationships that could be construed as a potential conflict of interest.

## Publisher’s note

All claims expressed in this article are solely those of the authors and do not necessarily represent those of their affiliated organizations, or those of the publisher, the editors and the reviewers. Any product that may be evaluated in this article, or claim that may be made by its manufacturer, is not guaranteed or endorsed by the publisher.
