# Supplementary material for: Associations of lipid parameters with non-alcoholic fatty liver disease in type 2 diabetic patients according to obesity status and metabolic goal achievement
Source: Front Endocrinol (Lausanne). 2022 Sep 16;13:1002099. doi: 10.3389/fendo.2022.1002099 (PMC9523101; doi:10.3389/fendo.2022.1002099)
Supplement: Supplementary file 1 [file DataSheet_1.docx]

Supplementary Table 1 Odds ratios and 95% confidence intervals of lipid parameters for non-alcoholic fatty liver disease according to obesity status.

|  | Model | Total | Obese | Non-obese |
| --- | --- | --- | --- | --- |
| TC | 1 | 1.31 (1.19-1.45)^*^ | 1.10 (0.82-1.47) | 1.34 (1.20-1.50)^*^ |
|  | 2 | 1.25 (1.12-1.39)^*^ | 1.10 (0.75-1.37) | 1.31 (1.16-1.48)^*^ |
|  | 3 | 1.27 (1.13-1.45)^*^ | 1.04 (0.77-1.42) | 1.33 (1.16-1.52)^*^ |
|  | 4 | 1.25 (1.11-1.40)^*^ | 1.05 (0.77-1.43) | 1.29 (1.12-1.47)^*^ |
| TG | 1 | 1.30 (1.22-1.38)^*^ | 1.12 (0.94-1.33) | 1.29 (1.21-1.39)^*^ |
|  | 2 | 1.21 (1.14-1.29)^*^ | 1.10 (0.92-1.30) | 1.23 (1.14-1.32)^*^ |
|  | 3 | 1.20 (1.12-1.27)^*^ | 1.13 (0.94-1.36) | 1.20 (1.12-1.29)^*^ |
|  | 4 | 1.19 (1.12-1.27)^*^ | 1.12 (0.93-1.35) | 1.20 (1.12-1.29)^*^ |
| HDL-C | 1 | 0.14 (0.09-0.22)^*^ | 0.06 (0.01-0.33)^*^ | 0.16 (0.10-0.26)^*^ |
|  | 2 | 0.23 (0.18-0.37)^*^ | 0.06 (0.01-0.33)^#^ | 0.30 (0.18-0.51)^*^ |
|  | 3 | 0.21 (0.12-0.36)^*^ | 0.06 (0.01-0.33)^#^ | 0.27 (0.15-0.48)^*^ |
|  | 4 | 0.20 (0.11-0.34)^*^ | 0.08 (0.02-0.41)^#^ | 0.24 (0.13-0.43)^*^ |
| LDL-C | 1 | 1.19 (1.06-1.34)^#^ | 1.05 (0.73-1.50) | 1.24 (1.09-1.41)^*^ |
|  | 2 | 1.14 (1.00-1.30) | 0.91 (0.61-1.36) | 1.19 (1.03-1.38)^#^ |
|  | 3 | 1.13 (0.98-1.39) | 0.91 (0.60-1.38) | 1.19 (1.02-1.39)^#^ |
|  | 4 | 1.08 (0.93-1.25) | 0.91 (0.60-1.39) | 1.12 (0.95-1.33) |
| TC/HDL-C | 1 | 1.56 (1.42-1.71)^*^ | 1.41 (1.07-1.86)^#^ | 1.55 (1.40-1.71)^*^ |
|  | 2 | 1.39 (1.26-1.54)^*^ | 1.25 (0.94-1.66) | 1.37 (1.22-1.52)^*^ |
|  | 3 | 1.42 (1.28-1.58)^*^ | 1.30 (0.97-1.75) | 1.37 (1.22-1.54)^*^ |
|  | 4 | 1.38 (1.24-1.53)^*^ | 1.28 (0.95-1.73) | 1.35 (1.21-1.51)^*^ |
| TG/HDL-C | 1 | 1.19 (1.14-1.25)^*^ | 1.11 (0.97-1.26) | 1.18 (1.12-1.25)^*^ |
|  | 2 | 1.13 (1.08-1.18)^*^ | 1.09 (0.94-1.25) | 1.13 (1.07-1.19)^*^ |
|  | 3 | 1.12 (1.07-1.18)^*^ | 1.12 (0.96-1.30) | 1.11 (1.06-1.17)^*^ |
|  | 4 | 1.12 (1.07-1.17)^*^ | 1.10 (0.95-1.29) | 1.12 (1.06-1.18)^*^ |
| LDL-C/HDL-C | 1 | 1.53 (1.39-1.71)^*^ | 1.43 (0.99-2.08) | 1.52 (1.34-1.73)^*^ |
|  | 2 | 1.36 (1.20-1.54)^*^ | 1.22 (0.82-1.82) | 1.33 (1.16-1.53)^*^ |
|  | 3 | 1.37 (1.20-1.57)^*^ | 1.27 (0.84-1.92) | 1.34 (1.16-1.55)^*^ |
|  | 4 | 1.35 (1.18-1.46)^*^ | 1.25 (0.82-1.90) | 1.30 (1.12-1.50)^*^ |
| Non-HDL-C | 1 | 1.50 (1.35-1.68)^*^ | 1.19 (1.88-1.63) | 1.53 (1.36-1.73)^*^ |
|  | 2 | 1.39 (1.23-1.56)^*^ | 1.09 (0.79-1.49) | 1.44 (1.26-1.64)^*^ |
|  | 3 | 1.42 (1.25-1.61)^*^ | 1.12 (0.81-1.55) | 1.47 (1.28-1.70)^*^ |
|  | 4 | 1.40 (1.23-1.58)^*^ | 1.12 (0.81-1.55) | 1.41 (1.22-1.63)^*^ |
| Non-HDL-C/HDL-C | 1 | 1.56 (1.42-1.71)^*^ | 1.41 (1.07-1.86)^#^ | 1.55 (1.40-1.71)^*^ |
|  | 2 | 1.39 (1.26-1.54)^*^ | 1.25 (0.94-1.66) | 1.37 (1.22-1.52)^*^ |
|  | 3 | 1.42 (1.28-1.58)^*^ | 1.30 (0.97-1.75) | 1.37 (1.22-1.54)^*^ |
|  | 4 | 1.38 (1.24-1.53)^*^ | 1.28 (0.95-1.73) | 1.35 (1.21-1.51)^*^ |

Model 1 was adjusted for age, sex, smoking status, family history of diabetes mellitus.

Model 2 was adjusted for all the variables in model 1 plus SBP, BMI, HbA1c and use of anti-hypertensive drugs for total; In obesity and non-obesity subgroup, BMI was replaced by waist circumference.

Model 3 was adjusted for all the variables in model 2 plus HOMA-IR.

Model 4 was adjusted for all the variables in model 3 plus use of anti-diabetic drugs.

TC, total cholesterol; TG, triglycerides; HDL-C, high density lipoprotein cholesterol; LDL-C, low density lipoprotein cholesterol; SBP, systolic blood pressure; BMI, body mass index; HbA1c, glycated hemoglobin.

^*^P < 0.001, ^#^P < 0.05.

Supplementary Table 2 Odds ratios 95% confidence intervals of lipid parameters for non-alcoholic fatty liver disease according to metabolic goal attainment status.

|  | HbA1c ≥ 6.5% | HbA1c < 6.5% | BP ≥ 130/80mmHg | BP <130/80mmHg | LDL-C ≥ 2.6mmol/L | LDL-C < 2.6mmol/L |
| --- | --- | --- | --- | --- | --- | --- |
| TC | 1.22 (1.07-1.41)^#^ | 3.31 (1.13-9.69)^#^ | 1.21 (1.05-1.40)^#^ | 1.38 (1.12-1.70)^#^ | 1.26 (1.04-1.54)^#^ | 1.38 (1.11-1.72)^#^ |
| TG | 1.21 (1.11-1.31)^*^ | 1.62 (1.07-2.47)^#^ | 1.26 (1.15-1.37)^#^ | 1.12 (1.03-1.22)^#^ | 1.38 (1.21-1.56)^*^ | 1.14 (1.07-1.22)^*^ |
| HDL-C | 0.25 (0.13-0.47)^*^ | 0.02 (0.001-0.39)^#^ | 0.14 (0.07-0.27)^*^ | 0.48 (0.17-1.30) | 0.21 (0.10-0.45)^*^ | 0.14 (0.06-0.32)^*^ |
| LDL-C | 1.06 (0.89-1.25) | 12.41 (2.01-73.37)^#^ | 1.01 (0.84-1.22) | 1.25 (0.96-1.64) | 1.22 (0.92-1.61) | 0.99 (0.61-1.59) |
| TC/HDL-C | 1.37 (1.22-1.55)^*^ | 3.46 (1.63-7.36)^#^ | 1.44 (1.27-1.64)^*^ | 1.27 (1.08-1.49)^#^ | 1.48 (1.27-1.74)^*^ | 1.31 (1.15-1.50)^*^ |
| TG/HDL-C | 1.14 (1.07-1.21)^*^ | 1.82 (1.18-2.79)^#^ | 1.16 (1.09-1.24)^*^ | 1.08 (1.01-1.15)^#^ | 1.34 (1.19-1.51)^*^ | 1.09 (1.04-1.14)^*^ |
| LDL-C/HDL-C | 1.28 (1.09-1.49)^#^ | 6.19 (2.00-19.15)^#^ | 1.37 (1.16-1.61)^*^ | 1.33 (1.05-1.68)^#^ | 1.51 (1.24-1.84)^*^ | 1.43 (1.08-1.90)^#^ |
| Non-HDL-C | 1.35 (1.16-1.56)^*^ | 2.17 (1.10-4.28)^#^ | 1.38 (1.17-1.61)^*^ | 1.44 (1.06-1.79)^*^ | 1.47 (1.20-1.81)^*^ | 1.58 (1.24-2.00)^*^ |
| Non-HDL-C/HDL-C | 1.37 (1.22-1.55)^*^ | 3.46 (1.63-7.36)^#^ | 1.44 (1.27-1.64)^*^ | 1.27 (1.08-1.49)^#^ | 1.48 (1.27-1.74)^*^ | 1.31 (1.15-1.50)^*^ |

Model for HbA1c subgroup was adjusted for age, sex, smoking status, family history of diabetes mellitus, SBP, BMI, RBG(random blood glucose), HOMA-IR, use of anti-hypertensive drugs, and anti-diabetic drugs;

Model for blood pressure subgroup was adjusted for age, sex, smoking status, family history of diabetes mellitus, BMI, HbA1c, HOMA-IR, use of anti-hypertensive drugs, and anti-diabetic drugs;

Model for LDL-cholesterol subgroup was adjusted for age, sex, smoking status, family history of diabetes mellitus, SBP, BMI, HbA1c, HOMA-IR, use of anti-hypertensive drugs, and anti-diabetic drugs.

TC, total cholesterol; TG, triglycerides; HDL-C, high density lipoprotein cholesterol; LDL-C, low density lipoprotein cholesterol; SBP, systolic blood pressure; BMI, body mass index; HbA1c, glycated hemoglobin; BP, blood pressure.

^*^P < 0.001, ^#^P < 0.05.
